# The interaction between integration and segmentation neurons for motion perception

**DOI:** 10.1186/1471-2202-16-S1-P86

**Published:** 2015-12-18

**Authors:** Parvin Zarei, Tatiana Kameneva, Michael R Ibbotson, Anthony N Burkitt, David B Grayden

**Affiliations:** 1 NeuroEngineering Lab, Dept Electrical & Electronic Engineering, University of Melbourne, Melbourne, VIC 3010, Australia; 2 NICTA, Sydney, Australia; 3 National Vision Research Institute, Australian College of Optometry, Melbourne, VIC 3053, Australia; 4 Bionics Institute, Melbourne, VIC 3002, Australia

## 

The main challenge for understanding motion processing in the visual system is that local motion signals do not accurately represent the direction of the whole object. This is known as the "aperture problem". The small receptive fields of individual neurons can only measure motion components orthogonal to their preferred direction. Neuro-physiological studies show that neurons in brain area MT (V5) have a major role in dealing with this problem [1]. We develop a model to overcome the aperture problem through excitatory and inhibitory interconnections between MT neurons.

The model contains two levels of rate-based neuron. The first level is a model of V1 with two sets of neurons: complex neurons and hyper-complex end-stopped neurons. End-stopped neurons respond to the end-points of the stimulus , thereby representing an unambiguous estimation of the direction of motion [2]. The response of V1 neurons is transmitted to the second level, the model of MT neurons. MT neurons are categorized as integration and segmentation neurons. The role of integration neurons is to integrate local motion signals to solve the aperture problem; the role of segmentation neurons is to detect discontinuities in the input stimulus to inhibit integration neurons [3].

Excitatory recurrent connections between integration neurons propagate end-stopped information from the object terminators. As each spatial location contains neurons selective to different directions, inhibitory connections are added between the neurons such that there will be only one neuron active at each spatial location. Long-range inhibitory connections are also added between more distant MT neurons selective to other directions. Segmentation neurons send inhibitory connections to integration neurons to prevent the propagation of motion signals outside the border of the input stimulus. Inhibitory connections between segmentation neurons are defined based on center-surround interactions; this suppresses activity when the motion signals in the center and surround are in the same directions; ensuring that their activity will not prevent the propagation of motion signals when there is no discontinuity.

The results of simulations show that the activity of V1 neurons along the edges is much stronger than the activity of the neurons at the end-points. Activation of end-stopped neurons in V1 suppresses ambiguous motion signals along the edges of the stimulus. Therefore, unambiguous information at the end-points wins the competition over the neurons with ambiguous motion signals. The final output of the model shows that MT neurons achieve an accurate estimation of motion using the information provided by the end-stopped neurons.

We developed a model of MT neurons for motion perception based on the available neuro-physiological data. These neurons receive their input from neurons in V1. The results show the successful function of these neurons in the perception of motion of a single moving bar. In this model, we also investigated the role of end-stopped neurons in overcoming the aperture problem. The results suggest that MT neurons are not able to solve the aperture problem without the vital role played by end-stopped cells.
